# Effect of Confining Conditions on the Hydraulic Conductivity Behavior of Fiber-Reinforced Lime Blended Semiarid Soil

**DOI:** 10.3390/ma14113120

**Published:** 2021-06-06

**Authors:** Abdullah Ali Shaker, Mosleh Ali Al-Shamrani, Arif Ali Baig Moghal, Kopparthi Venkata Vydehi

**Affiliations:** 1 Bugshan Research Chair in Expansive Soils, Department of Civil Engineering, College of Engineering, King Saud University, P.O. Box 800, Riyadh 11421, Saudi Arabia; shaker83@windowslive.com; 2 Department of Civil Engineering, National Institute of Technology, Warangal 506004, India; kvvydehi252@gmail.com

**Keywords:** confining pressure, expansive clay, fiber, flexible wall permeameter, hydraulic conductivity, lime, rigid wall permeameter

## Abstract

The hydraulic properties of expansive soils are affected due to the formation of visible cracks in the dry state. Chemical stabilization coupled with fiber reinforcement is often considered an effective strategy to improve the geotechnical performance of such soils. In this study, hydraulic conductivity tests have been conducted on expansive clay using two different types of fibers (fiber cast (FC) and fiber mesh (FM)) exhibiting different surface morphological properties. The fiber parameters include their dosage (added at 0.2% to 0.6% by dry weight of soil) and length (6 and 12 mm). Commercially available lime is added to ensure proper bonding between clay particles and fiber materials, and its dosage was fixed at 6% (by dry weight of the soil). Saturated hydraulic conductivity tests were conducted relying on a flexible wall permeameter on lime-treated fiber-blended soil specimens cured for 7 and 28 days. The confining pressures were varied from 50 to 400 kPa, and the saturated hydraulic conductivity values (k_sat_) were determined. For FC fibers, an increase in fiber dosage caused k_sat_ values to increase by 9.5% and 94.3% for the 6 and 12 mm lengths, respectively, at all confining pressures and curing periods. For FM fibers, k_sat_ values for samples mixed with 6 mm fiber increased by 12 and 99.2% for 6 and 12 mm lengths, respectively for all confining pressures at the end of the 28-day curing period. The results obtained from a flexible wall permeameter (FWP) were compared with those of a rigid wall permeameter (RWP) available in the literature, and the fundamental mechanism responsible for such variations is explained.

## 1. Introduction

Desiccation heave and shrinkage characteristics of expansive soils have limited their application as a subbase for roadway pavements material in arid and semiarid climates. Randomly oriented fibers are often used to reduce the problems associated with clayey soils. The cost-effectiveness and chemical inertness of the fibers makes them useful as a soil additive [1,2]. To develop the bonding between soil grains, researchers used chemical stabilizers such as lime, cement, EICP, MICP, biopolymer, etc., in combination with fiber to improve the geotechnical properties [3,4,5,6]. The addition of fiber to the soil increases shear strength [7], reduces swelling [8,9], reduces desiccation cracking [10], and increases hydraulic conductivity (k) values [11,12]. Although fiber inclusion reduces the desiccation cracking, an increase in k values of soil limited its application in the subbase for the construction of pavements. However, the resultant properties of fiber-reinforced soil depend on the fiber type, dosage, and aspect ratio [13]. Experimental investigations on medium plasticity clay with polypropylene fiber (at 2%) inclusion results in an increase in k values by 10^−3^ cm/s compared to untreated clay. The fiber dosage up to 0.5% resulted in acceptable hydraulic conductivity values [12]. On the contrary, k values of lime-amended high-plastic clay reduced by 10^−2^ cm/s with a fiber dosage of 0.2% and length of 6 mm at the end of a 28-day curing period compared to specimens without curing [14]. To meet the requirements of hydraulic conductivity values for lime-stabilized high-plastic clay reinforced with fiber, an optimum length of 10.5 mm with 0.5% dosage for a 15-day curing period is proposed using the response surface method [15]. On the other hand, few researchers have studied the effect of lime on the hydraulic conductivity of soils. The results showed an increase in the hydraulic conductivity with lime [16,17,18]. In addition, the k value of lime-treated soil increased at initial curing periods and then decreased at higher curing periods [19,20]. Earlier research concentrated on evaluating the hydraulic conductivity of fiber-reinforced soil using a conventional rigid wall permeameter (RWP).

However, for a compacted clay as a subgrade layer, the surcharge load coming on it plays a major role in controlling the hydraulic conductivity values. According to Daniel et al. [21], complete control over imposed stress on the soil is not possible, resulting in the inability of RWP to measure vertical and horizontal deformations. To overcome these limitations, a flexible wall permeameter (FWP) was introduced, in which back pressure saturation and the minimization of sidewall leakages facilitate in determining the accurate value of saturated hydraulic conductivity values (k_sat_) [22]. Therefore, to simulate the real field conditions, researchers proposed using FWP to evaluate k_sat_ values. Experimental investigations on sand–Alqatif clay mixture revealed that k_sat_ values reduced with an increase in confining pressure [23]. The generalized mechanism proposed for the reduction of k_sat_ values using FWP is that an increase in confining pressure reduces the effective pore spaces, and an increase in unit weight leads to a reduction of effective flow paths.

There are limited studies on the evaluation of k_sat_ values for fiber-reinforced soil using FWP. In lieu of this, the present research evaluates the k_sat_ values of a lime-stabilized Al-Ghat soil with fiber inclusion using FWP. The effect of dosage, length of the fiber, and curing period are considered in evaluating the k_sat_ values. Then, the values obtained from the study are compared with the values in the literature [13] to evaluate the effectiveness of FWP in the accurate measurement of k_sat_ values.

## 2. Materials

### 2.1. Soil

Locally available natural soil sourced from Al Ghat (26°1′36″ N, 44°57′39″ E) town, Riyadh Province, Saudi Arabia, was selected for the present study. Disturbed samples were obtained from open test pits excavated to a depth of 1.5–3.0 m below the ground surface. The physical characterization of soil (carried out in accordance with relevant ASTM standards) seen from Table 1 reveals that the selected soil is a high plastic clay (CH) as per the Unified Soil Classification System (USCS), and it is expansive in nature [13].

### 2.2. Fiber

**Figure 1:**
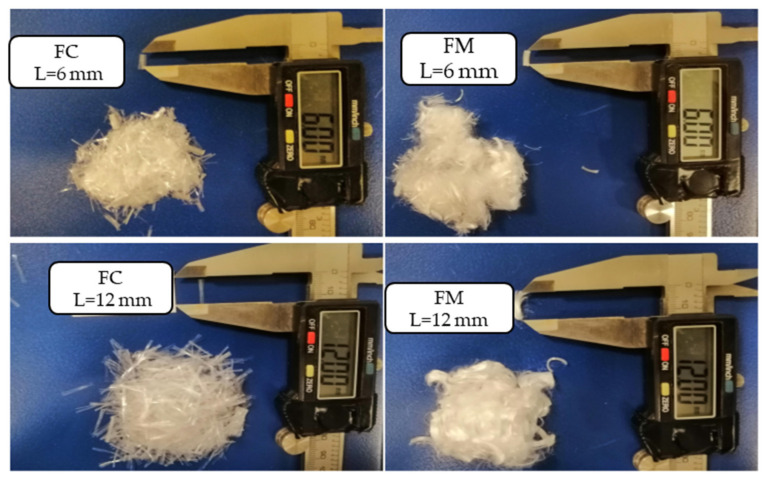
Fibers used in the study.

Two types of fibers FM 300 and FC 500 supplied by Propex operating company (Chattanooga, TN, USA) were used for the present study. The physicochemical properties of two fibers are provided in Table 2. The length of fiber adopted for the experimental work is 6 mm and 12 mm for both FM and FC (Figure 1); the dosage of each fiber is fixed at 0.2% and 0.6% by weight of dry soil mass.

### 2.3. Lime

Locally available commercial-grade hydrated lime is used as a soil stabilizer. The percentage of lime was standardized at 6% for all tests by dry weight of the soil. This optimum quantity of lime was fixed relying on the soil–pH response upon the addition of lime [14,24].

## 3. Experimental Program

### 3.1. Sample Preparation

The expansive soil obtained from the field was air-dried, pulverized, and sieved using sieve No. 20. The soil fraction is mixed with optimum lime content (i.e., 6% of lime added to the soil by dry weight) and fiber (FC and FM) at selected dosages of 0.2% and 0.6%. The lengths of fibers were kept at 6 mm and 12 mm, resulting in 17 mix combinations in triplicates (including untreated soil) for the entire experimental work. A total of 51 specimens (including triplicates) were tested in FWP. Previous studies on the compaction properties of fiber-reinforced soil have revealed that the fiber inclusion had little or no effect on variations in maximum dry density (MDD) and optimum moisture content (OMC) of various mix proportions [12,25,26]. Based on this, all the specimens in the present study were molded at fixed MDD (16.08 kN/m^3^) and corresponding OMC (25%). Soil, lime, and fibers at various proportions were mixed in dry condition; then, the target moisture content was added and mixed thoroughly to obtain a homogenous mixture. Statically compacted specimens of 70 mm diameter and 35 mm height were kept in a desiccator at a relative humidity > 95% and cured for a period of 7 and 28 days.

### 3.2. Testing Procedure

**Figure 2:**
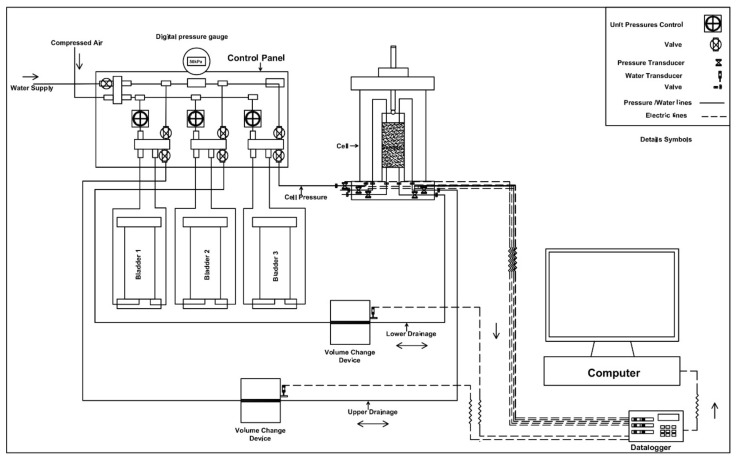
Schematic diagram of a flexible wall constant head permeameter.

The evaluation of saturated hydraulic conductivity (k_sat_) was performed in accordance with ASTM D5084—Method A [27], using a flexible wall constant head permeameter. A schematic diagram of the test setup used in the present study is shown in Figure 2. At the end of each curing period, the prepared specimens were transferred to the cell. Porous stone and filter paper were kept on the top and bottom end of the specimen. A rubber membrane was used to confine the specimen; two O-rings placed at both ends provide a complete seal against any water leakage. The cell was filled with distilled water, and the drainage line at the bottom and top of the cell was flushed until no more air bubbles were observed inside the cell. The various stages involved in the testing phase are described below.

I. Back-Pressure Saturation:

This stage involved a simultaneous increase of both the cell pressure (CP) and the back pressure (BP) to reduce air bubbles or voids within the test sample. In this study, the effective confining pressure (defined as cell pressure minus back pressure, CP-BP) was kept at approximately 10 kPa throughout the saturation process for all specimens. This effective confining pressure was selected to maintain sample stability without significantly affecting the stress history of the specimen. The (CP-BP) was maintained for one day. Specimen saturation was verified by measuring the B coefficient (defined as the difference in pore-water pressure (Δu), divided by the difference in pressure of the cells (ΔCP) of the porous material). A saturation check involved increasing the pressure of the cell on the specimen and monitoring the pore pressure response using a pore pressure transducer connected at the top and bottom of the specimen. The theoretical B value for a fully saturated specimen reaches 1. However, in fluid flow experiments, specimens were considered saturated with the assurance of B values ≥ 0.95. If the B value is less than 0.95, the above procedure of increasing CP and BP and B value checking was repeated until the B value is >0.95.

II. Consolidation:

The specimens were consolidated under effective confining pressure (CP-BP) of 50, 100, 200, and 400 kPa. Effective confining pressure was applied by increasing the cell pressure to the level necessary to develop the desired effective confining pressure while maintaining a constant back pressure. Drainage was allowed from the base of the specimen. The outflow volumes were recorded to confirm that primary consolidation has been completed before the initiation of the next stage.

III. Permeation:

This stage involved inducing flow-through test specimens by applying a differential pressure between the top and bottom of the specimens. The differential pressure was applied by reducing the top pressure and increasing the bottom pressure such that the difference was equal to the pressure head corresponding to the desired hydraulic gradient. To speed up the test, the hydraulic gradient was fixed at 30 [27]. The water inflow and outflow were continuously monitored until a steady-state condition was established as defined by the inflow rate being equal to the outflow rate.

## 4. Results and Discussion

### 4.1. Effect of Confining Pressure

**Figure 3:**
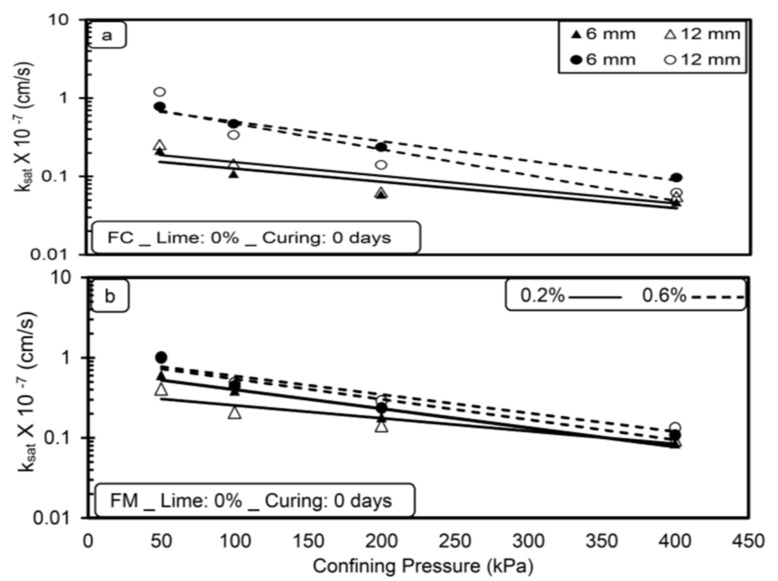
Variations in saturated hydraulic conductivity (k_sat_) with confining pressure (**a**) FC (**b**) FM without lime treatment (without curing).

**Figure 4:**
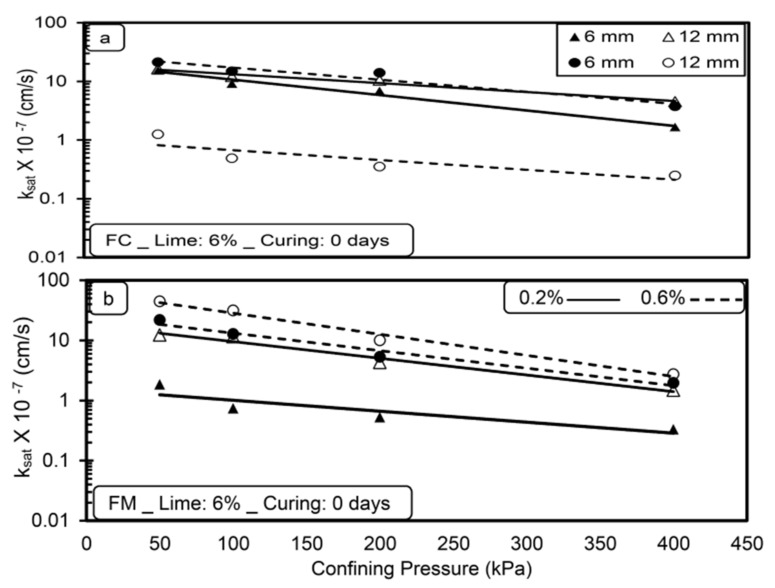
Variations in saturated hydraulic conductivity (k_sat_) with confining pressure (**a**) FC (**b**) FM with lime treatment (without curing).

**Figure 5:**
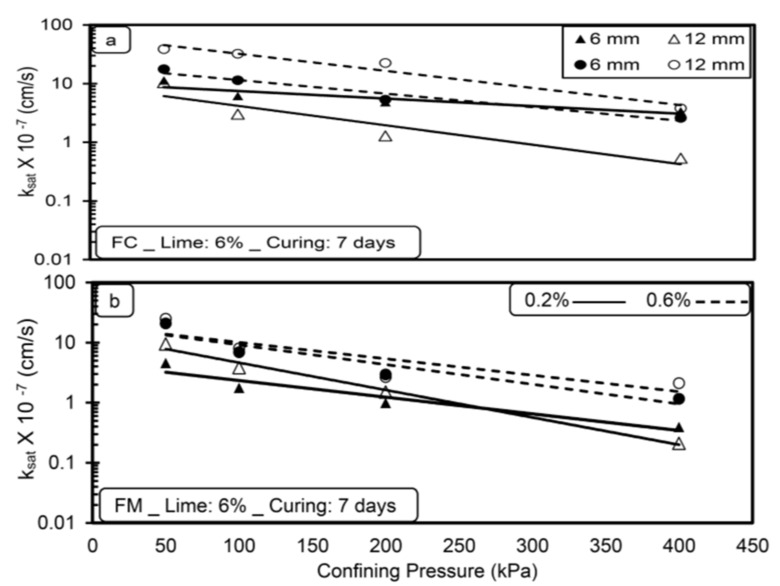
Variations in saturated hydraulic conductivity (k_sat_) with confining pressure (**a**) FC (**b**) FM with lime treatment (after 7-day curing period).

**Figure 6:**
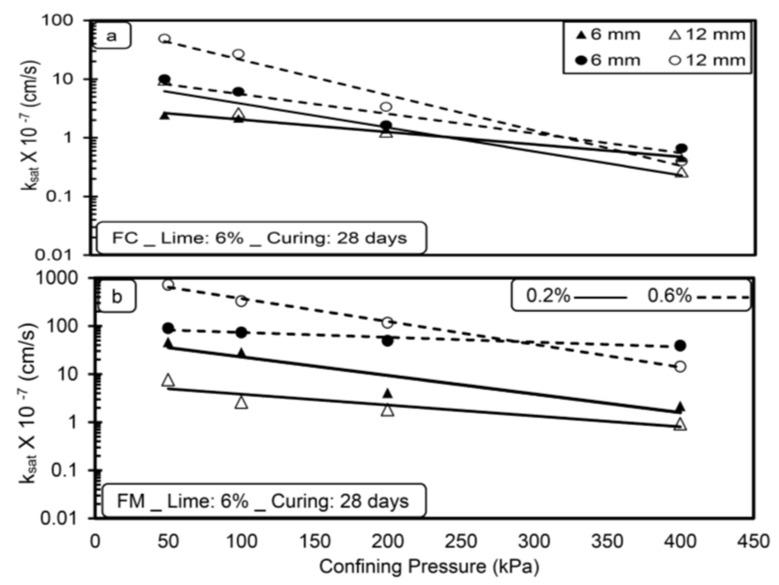
Variation of saturated hydraulic conductivity with confining pressure (**a**) FC (**b**) FM with lime treatment (after 28-day curing period).

Figure 3, Figure 4, Figure 5 and Figure 6 show the variation in saturated hydraulic conductivity (k_sat_) values for lime-treated (at 6%) expansive soil with fiber inclusion (0.2% and 0.6%) at various effective confining pressures at the end of each curing period.

In general, the k_sat_ values reduced with an increase in confining pressure for all the tested specimens irrespective of fiber type, dosage, and curing period. A noticeable reduction in k_sat_ values is observed when the confining pressure is increased from 50 to 200 kPa. The flow of water through the compacted specimen depends on the availability and connectivity of inter and intra-aggregate flow channels, and the k value is directly related with inter-aggregate flow paths [28,29]. Increased confining pressure contributes to a significant reduction in inter-aggregate flow paths compared to intra-aggregate flow paths. Due to this, with the increase in pressure from 50 to 200 kPa, a significant reduction in inter-aggregate flow channels causes a decrease in k_sat_ values. A further increment in confining pressure from 200 to 400 kPa has less effect in reducing these flow paths and leads to a marginal reduction in k_sat_ values for all the tested specimens.

For any type of soil, the higher the confining pressure, the lower the k_sat_ values, irrespective of the permeating liquid [23]. Increased confinement causes a reduction in pore spaces and increases the unit weight, thus reducing the hydraulic conductivity [30]. Similar results were reported by de Brito Galvão et al. [31] and Shaker and Elkady [32].

The boundary condition adopted in the present study is highly correlated with the field conditions for the case of a subbase for pavements material in which the subbase material will be subjected to surcharge load coming on it.

### 4.2. Effect of Lime

**Figure 7:**
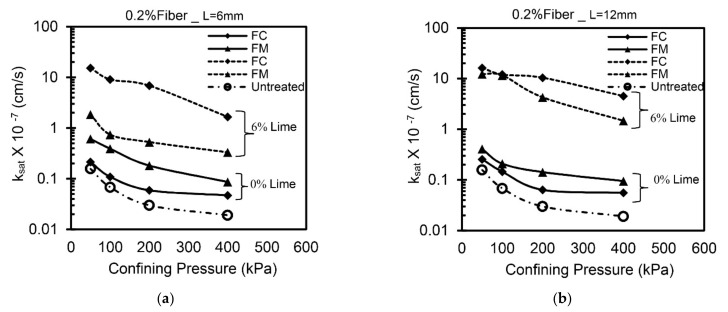

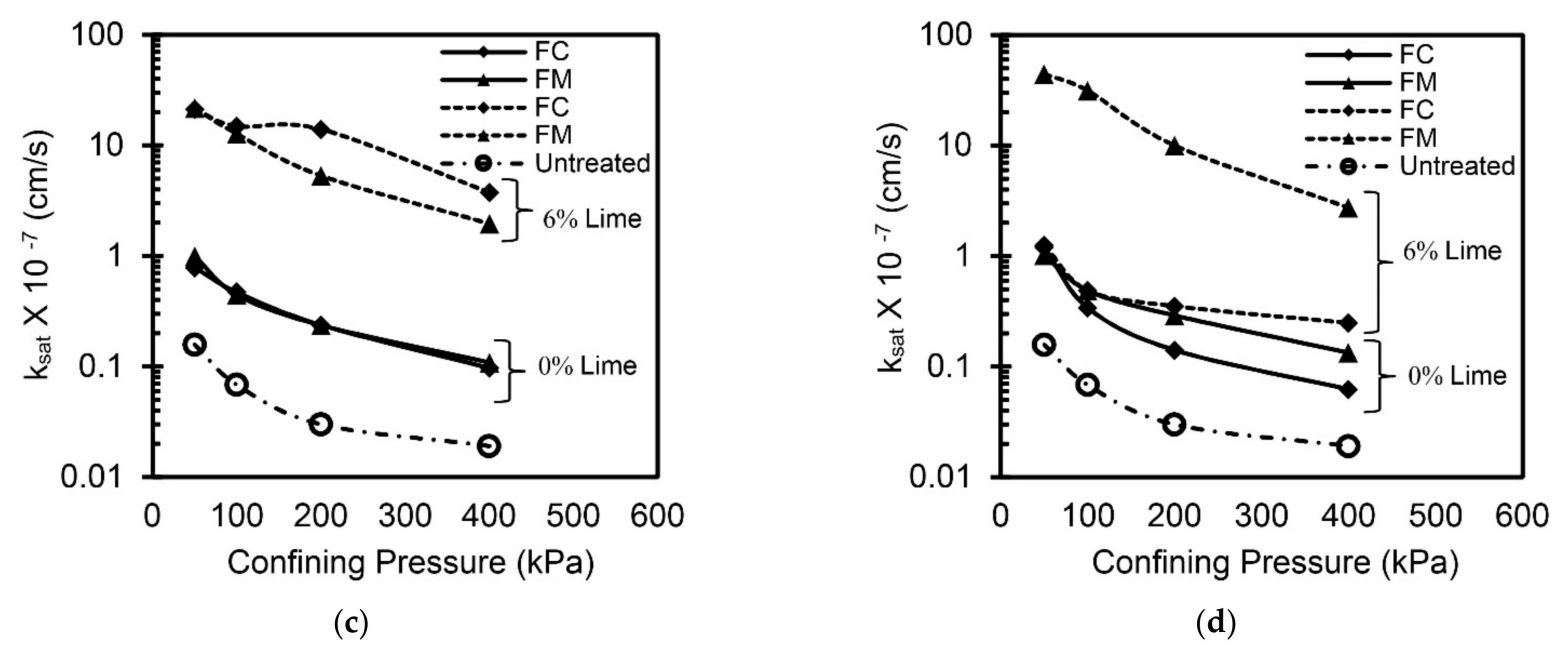
Variation of saturated hydraulic conductivity with lime content and confining pressure (**a**) 0.2%, 6mm; (**b**) 0.2%, 12mm; (**c**) 0.6%, 6mm; (**d**) 0.6%, 12mm.

Figure 7 shows the variations in (k_sat_) values with and without lime treatment for fiber-reinforced clay (FC and FM) without any curing. The addition of 6% lime causes an increase in k_sat_ values in the order of 10^−1^ cm/s for fiber-reinforced clay compared to that of an untreated case. The addition of lime leads to the aggregation of soil grains by Ca^2+^ ions, resulting in the formation of a flocculated structure [33,34]. The increased porosity of soil improves the connectivity of inter-aggregate pores and leads to an increase in k_sat_ values [31]. A similar trend is observed for all the tested specimens; however, the rate of increase in k_sat_ values is a function of fiber type, dosage, and length.

### 4.3. Effect of Curing Time

**Figure 8:**
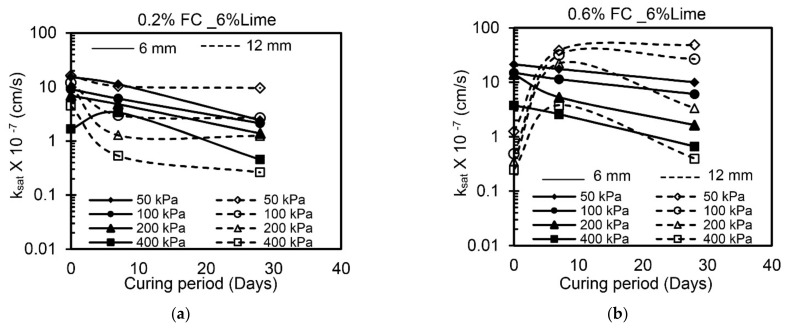

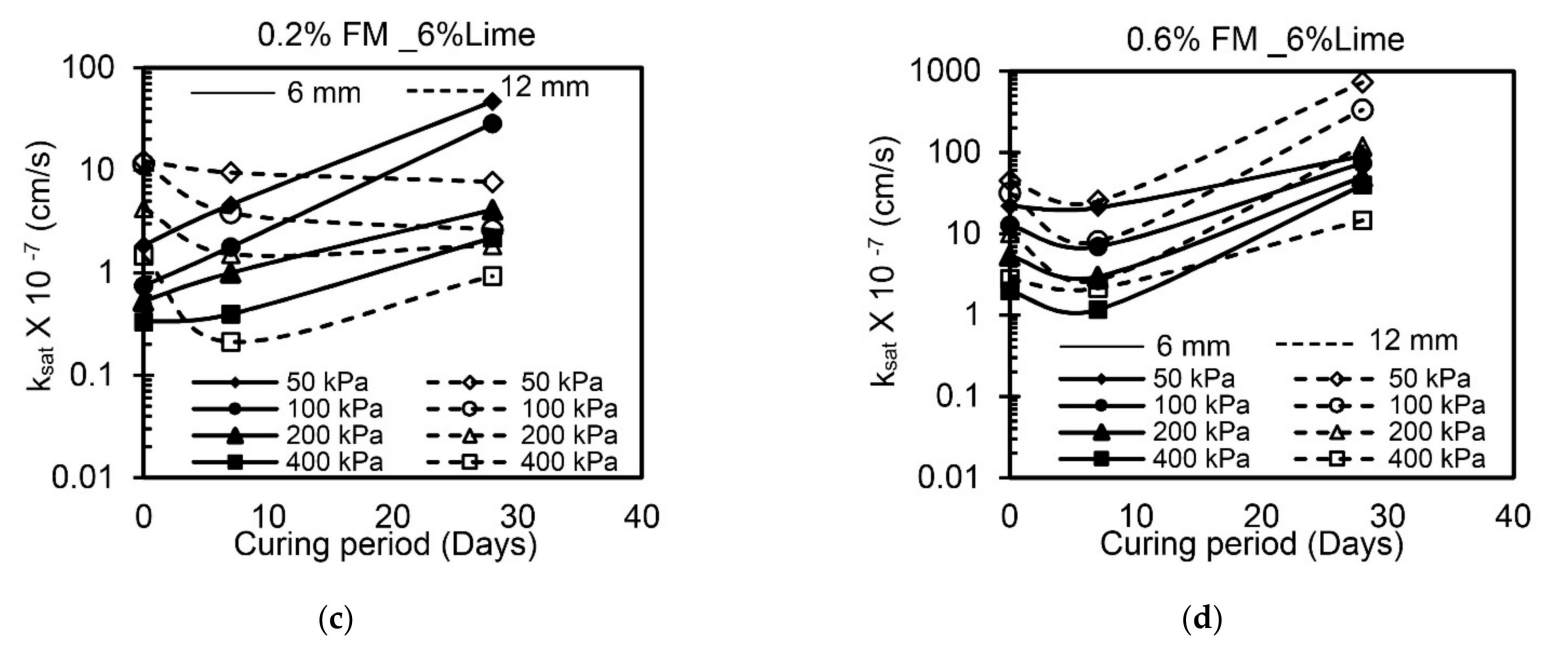
Variation of k_sat_ values with curing period and confining pressure (**a**) FC_0.2% (**b**) FC_0.6% (**c**) FM_0.2% (**d**) FM_0.6%.

The effect of the curing period on the k_sat_ values of all samples with lime-treated expansive clay under different confining pressures is illustrated in Figure 8. Lime-treated soil reinforced with 0.2% FC (both 6 mm and 12 mm) exhibited a reduction in k_sat_ values with an increase in curing period, as seen from Figure 8a. This is attributed to the fact that the cementitious compounds formed at a higher curing period fill the void spaces within the clay and the soil becomes less conductive [13]. At 0.6% FC, for 6 mm length of fibers, k_sat_ values reduced marginally at a lower confining pressure (at 50 kPa), and a significant reduction is observed at higher confining pressure (at 400 kPa). Whereas, an increase in fiber length to 12 mm leads to an increase in k_sat_ values up to 7 days of curing and thereafter reduced at the end of a 28-day curing period (Figure 8b). This might be due to an increase in the length of fiber creating more drainage paths during the first 7 days of curing. However, after a 28-day curing period, this effect is dominated by the formation of cementitious compounds, which reduces the k_sat_ values with 12 mm fiber length.

Figure 8c,d depict the variations in k_sat_ values with FM addition at various curing periods. The k_sat_ values increased at the end of the 28-day curing period with a marginal reduction at 7 days compared with specimens without curing. Irrespective of the dosage and length of FM, the k_sat_ values increased at all confining pressures.

### 4.4. Effect of Fiber

The results of permeability tests indicate that the k_sat_ is a function of fiber type, dosage, and length (Figure 8). With an increase in dosage from 0.2 to 0.6%, the k_sat_ values increased irrespective of the type of fiber for all specimens and are attributed to the randomly distributed fibers increasing the flow paths and causing free movement of the permeating liquid. In addition, an increase in fiber length from 6 to 12 mm results in an increase in k_sat_ values. However, this effect is not significant at higher curing periods. Relatively, the inclusion of FM results in increased k_sat_ values compared to the inclusion of FC, especially at 0.6% dosage. This behavior is attributed to the fact that the FM has a rough surface with protrusions (Table 2) compared to FC, which facilitates in creating more drainage paths and leading to an increase in the resultant k_sat_ values; whereas FC having a relatively smooth texture offers fewer drainage paths and leads to a reduction in k_sat_ values at higher curing periods. Similar observations were reported by Abdi et al. [10] and Maher and Ho [11].

### 4.5. Comparison between Flexible Wall Permeameter and Rigid Wall Permeameter

**Figure 9:**
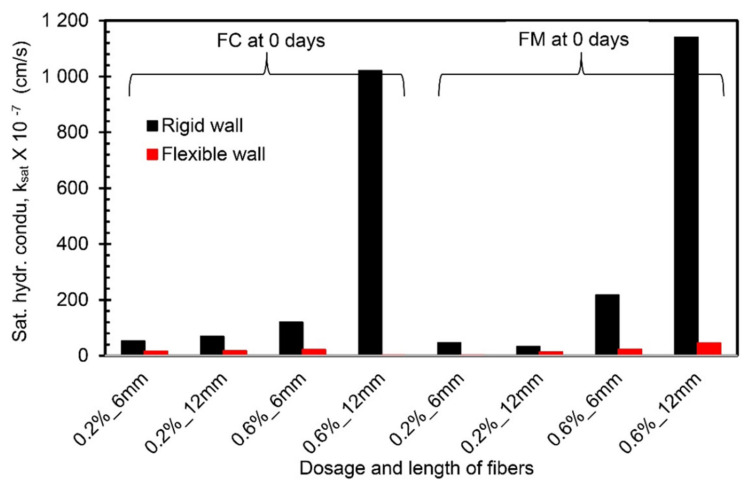
Comparison of hydraulic conductivity values from FWP and RWP (0-day curing period).

**Figure 10:**
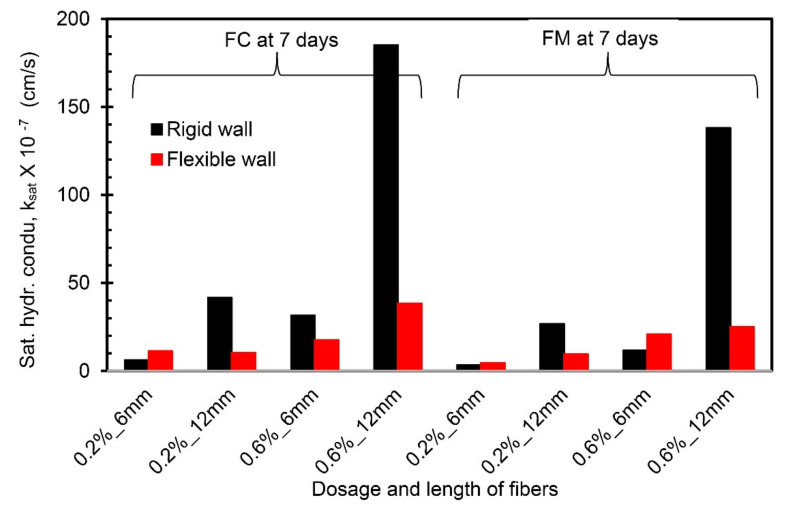
Comparison of hydraulic conductivity values from FWP and RWP (7-day curing period).

**Figure 11:**
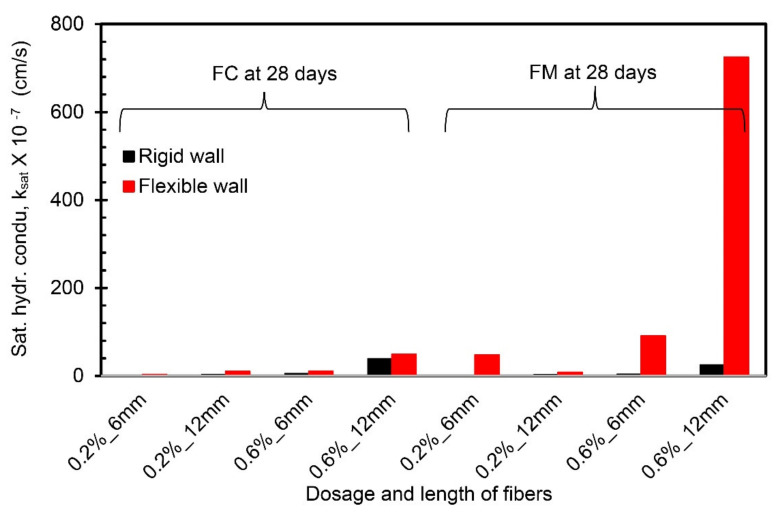
Comparison of hydraulic conductivity values from FWP and RWP (28-day curing period).

A comparison of the k_sat_ values obtained from the present study (using FWP at 50 kPa) and Moghal et al. [13] (using RWP) is presented in Figure 9, Figure 10 and Figure 11 for the same materials under the same testing conditions. From Figure 9 and Figure 10, the k_sat_ values obtained from FWP are lower than the k_sat_ values obtained from RWP tests for the fiber-reinforced lime-treated soil specimens up to a 7-day curing period irrespective of fiber type. A notable variation in k_sat_ values (in the order of 10^−1^) is observed for specimens with 0.6% dosage and 12 mm length of fiber (FC and FM) compared to other combinations. Since complete control over the confining pressure and back saturation of a specimen prior to testing is possible in FWP, it leads to an accurate measurement of vertical and horizontal deformations and thus k_sat_ values [21]. From the comparison, it is understood that FWP gives reliable results simulating the prevailing conditions in the field. For specimens cured for 28 days, the values of k_sat_ obtained using FWP are higher than those obtained from RWP (Figure 11). Experimental results on RWP have revealed that at higher curing periods (28 days), the leakage of liquid through the sidewalls of RWP due to loss of soil contact may significantly influence the k_sat_ values.

## 5. Conclusions

The present study evaluated the effect of fiber inclusion (FC and FM) on saturated hydraulic conductivity (k_sat_) of lime-treated expansive clay. The constant head method using FWP at various confining pressures is adopted for experimental work. The k_sat_ values were evaluated at various dosages (0.2% and 0.6%), fiber lengths (6 and 12 mm), and curing periods (7 and 28 days). The following conclusions are drawn:

- The addition of lime significantly increased the k_sat_ values compared to untreated specimens.
- At lower confining pressures (50 to 200 kPa), the reduction in k_sat_ values is attributed to a decrease in inter-aggregate flow paths. This effect is not significant at higher confining pressure (400 kPa).
- An increase in the dosage and lengths of fiber leads to an increase in k_sat_ values irrespective of the fiber type.
- For FC at 0.2%, the k_sat_ values reduced at the end of the 28-day curing period irrespective of the length of fiber used. At 0.6% dosage, the values marginally increased for the 12 mm fiber length due to increased drainage paths.
- For FM, the k_sat_ values increased at the end of 28 days irrespective of dosage and fiber length at all confining pressures.
- k_sat_ values are directly dependent on the nature and type of fiber used, of which FM fibers with extended protrusions enable higher friction levels. For FC fibers, increased fiber dosage from 0.2 to 0.6% has caused an increase in k_sat_ values by 9.5% and 94.3% for the 6 and 12 mm lengths, respectively, at all confining pressures. In contrast, for a similar change in fiber amount for FM fibers, the k_sat_ values for samples mixed with 6 mm fiber increased by 12 and 99.2% for 6 and 12 mm lengths, respectively for all confining pressures.

## Figures and Tables

**Table 1:** Physical properties of studied soil.

Physical Property	Value
Liquid Limit (%)	66
Plastic Limit (%)	32
Plasticity Index (%)	34
Shrinkage Limit (%)	15
Linear Shrinkage (%)	31
% Finer No.200 sieve	87
Natural Moisture Content (%)	3.2
Maximum Dry Density (kN/m^3^)	16.08
Optimum Water Content (%)	25
Specific Gravity	2.85
Specific Surface Area (BET Method) (m^2^/g)	27.08
USCS Classification	CH
Color	light brown

**Table 2:** Physicochemical properties of fibers.

Property	Fiber Cast 500 (FC)	Fiber Mesh 300 (FM)
Tensile Strength	440 N/mm^2^	330 N/mm^2^
Specific Gravity	0.91	0.91
Electrical Conductivity	Low	Low
Acid and Salt Resistance	High	High
Melt Point	324 °F (162 °C)	324 °F (162 °C)
Ignition Point	1100 °F (593 °C)	1100 °F (593 °C)
Thermal Conductivity	Low	Low
Water Absorption	Nil	Nil
Alkali Resistance	Alkali Proof	Alkali Proof
Surface Texture	Relatively Smooth	Rough with Protrusions
Shape	Fibrillated	Monofilament
Aspect Ratio (L/D)	42.8 and 120	193.5 and 387

## Data Availability

The content presented here was sourced from existing published literature, hence, this clause is not applicable.

## Abbreviations

The following symbols are used in this paper:

B	Coefficient of Saturation
BP	Back Pressure
CP	Cell Pressure
FC	Fiber Cast
FM	Fiber Mesh
FWP	Flexible Wall Permeameter
k_sat_	Saturated hydraulic conductivity
RWP	Rigid Wall Permeameter
ΔCP	The difference in cell pressure
Δu	The difference in pore-water pressure
